# Effective utilisation of National Rural Health Mission flexi-fund in Jharkhand: facilitators, barriers and options

**DOI:** 10.1186/1753-6561-6-S1-P7

**Published:** 2012-01-16

**Authors:** Suranjeen Prasad, Sapna Surendran, Tapan Chakraborty, Supriya Minz, Avinash Ansingkar, Arti Bhanot, Rajan Kumar

**Affiliations:** 1 Public Health Resource Network, Jharkhand, India; 2 Child In Need Institute, Ranchi, Jharkhand, India; 3 MCH-STAR (USAID), India; 4 State Programme Management Unit, Jharkhand, India

## Introduction

The National Rural Health Mission (NRHM) provides flexi-fund (a financing mechanism enabling pooling of money from fixed budget heads to be flexibly used as per local needs) to states and districts for paying for urgent but discreet expenses pertaining to maintenance of health infrastructure and provision of services at district, block and village level.

Decisions on use of such fund is to be made locally through various bodies/committees at district, block and village levels such as District Health Societies (DHS), Rogi Kalyan Samitis (RKS), Panchayati Raj Institutions (PRIs), Village Health and Sanitation Committees (VHSC) and village level health and Integrated Child Development Services (ICDS). Various policy documents and guidelines are available on use of flexi-fund.

Primarily, this fund is meant to ensure that health institutions at all the levels for healthcare services have readily available fund to overcome any bottlenecks that arise in the delivery of public health services. Amount of the flexi-find at various levels of healthcare services has been fixed (Table 1).

**Table 1:** Allocation of flexi-fund per year at various levels of healthcare services

Level of healthcare services	Flexi-fund INR (USD)		
	
	Untied Fund	Annual maintenance Grants (AMG)	RKS
District	NA	NA	5,00,000 (10736.5)
Block (community health centres)	50,000 (1073.7)	1,00,000 (2147.3)	1,00,000 (2147.3)
Block (primary health centres)	25,000 (536.8)	50,000 (1073.7)	1,00,000 (2147.3)
Village (health sub-centres)	10,000 (214.7)	10,000 (214.7)	NA
Village (VHSC)	10,000 (214.7)	NA	NA

As per the financial monitoring reports available from the state government, the utilisation of AMGs at primary health centres and community health centres level has remained around 50% in years 2008-09 and 2009-10. Utilisation of untied fund at health sub-centre level has been about 30% during the same years. Utilisation of untied fund at the village level by VHSCs has remained low.

We conducted this study to identify factors that impede or facilitate utilisation of flexi-fund at district and sub-district level. We aimed to use the findings of this study to developed revised operational guidelines on use of flexi-fund for consideration by state government.

## Methods

We reviewed relevant literature and used lessons from our work (using problem-solving approach) with health services to understand issues affecting underutilisation of flexi-fund in Jharkhand.

## Results

We observed that late release of fund and lack of clarity on decision-making processes as well as operational guidelines in regard to utilisation of flexi-fund were barriers to effective utilisation of flexi-fund. Other issues included poorly defined auditing as well as supervision/monitoring systems and lack of orientation to fund managers.

**Table 2:** Barriers related to underutilisation of specific type of flexi-fund

Level	Type of fund	Barriers to utilisation of flexi-fund
District and block	RKS	Formation and registration of district level societies Lack of clarity for technical and management Units
Block	AMG	Lack of clarity on accountability and role of managers in fund utilisation
Village (health sub-center)	AMG, Unitied fund	Lack of operational guideline Frontline health workers are new to fund management Guidelines for utilisation of fund prescribe specific items (against the principle of ‘untied’ fund)
Village (VHSC)	Untied fund	Delayed formation and registration of VHSCs Absence of PRI in the state Frontline health workers and VHSC members are new to fund management

## Discussion

Our findings suggest that existing guidelines for use of flexi-fund need revision in order to make them comprehensible and useful for fund managers and users. Any revisions in programme guidelines/implementation should be followed up to assess the appropriateness and effectiveness of revisions.

There is need to build capacity of fund managers especially as many of the staff appointed as fund managers are dealing with fund management first time. There is need to strengthen monitoring and evaluation systems. Despite political and socio-economic variations across the country, there is tremendous scope for adapting existing best practices from other states.

